# On the Dressing of Amputated Limbs

**Published:** 1855-08

**Authors:** 


On the Dressing of Amputated Limbs. By M. Coste.— In this paper, M. Coste, who is senior surgeon to the Hôtel Dieu at Marseilles, describes the mode of treating amputated limbs by what he terms *pansements rares*, or infrequent dressing. He alludes to M. Chaissaignac's mode of dressing by occlusion, which might seem to bear some resemblance to his own, and which consists in carefully closing up the compound fractures and other severe lesions by imbricated strapping, which is retained without changing for a week or fortnight. This is however, especially applicable to severe injuries of bone, where absolute quietude and the exclusion of air are important; and the loading a stump with so thick a covering of strapping would be a very questionable procedure. M. Sédillot's mode of dressing stumps, again, differs also from the author's. In fact, it consists in suppressing all dressing, a large anterior flap covering the surface of the wound by its own weight. A piece of rag about two finger's breadth, and spread with ointment, is folded up and applied around the end of the bone, so as to act as a canal for the discharge of the fluids. The angles of the flap are united by two points of suture, and the stump remains uncovered upon the napkin it is to lie upon, and which receives the pus as it escapes. The folded compress applied to the end of the bone is removed on the third or fourth day.

This mode is not applicable to the case of circular amputation, while that advocated by M. Coste may be employed in all modes. After the bleeding is arrested, the lips of the wound are brought gently together by two or three strips of plaster, taking care to leave the angles somewhat open for the passage of the ligatures and the escape of the fluids. Over this are applied a pledget, some layers of charpie, and a bandage. The first dressing only takes place from the sixth to the twelfth day after the operation, and in others from the eighth to the twelfth day; the occurrence of severe pain, hæmorrhage, sense of constriction or too abundant suppuration (all of which are extremely rare) indicating a condition of phlegmasia and the necessity for more frequent dressing. The two chief perils after amputation are inflammatory strangulation of the stump and retention of the pus, and M. Coste attributes the non-occurrence of the former in his stumps to the fact that he never employs sutures. He appeals to those who have witnessed his hospital practice as to whether rapid union and the production of a good stump are not the consequences of this mode of dressing.—*Med. Chir. Rev.*

